# Treatment principles in the management of open fractures

**DOI:** 10.4103/0019-5413.43373

**Published:** 2008

**Authors:** William W Cross, Marc F Swiontkowski

**Affiliations:** Department of Orthopaedic Surgery, University of Minnesota Medical School, 2450 Riverside Ave., Ste R 200, Minneapolis, MN 55454, USA

**Keywords:** Fracture principles, open fractures, trauma

## Abstract

The management of open fractures continues to provide challenges for the orthopedic surgeon. Despite the improvements in technology and surgical techniques, rates of infection and nonunion are still troublesome. Principles important in the treatment of open fractures are reviewed in this article. Early antibiotic administration is of paramount importance in these cases, and when coupled with early and meticulous irrigation and debridement, the rates of infection can be dramatically decreased. Initial surgical intervention should be conducted as soon as possible, but the classic 6 h rule does not seem to be supported in the literature. All open fractures should be addressed for the risk of contamination from *Clostridium tetani*. When possible, early closure of open fracture wounds, either by primary means or by flaps, can also decrease the rate of infection, especially from nosocomial organisms. Early skeletal stabilization is necessary, which can be accomplished easily with temporary external fixation. Adhering to these principles can help surgeons provide optimal care to their patients and assist them in an early return to function.

## INTRODUCTION

It has been estimated that between 3.5 and 6 million fractures occur in the United States annually.^1^,^2^ Extrapolating from European data, we can estimate that more than 3%, i.e., 150,000, of these are open fractures.^3^,^4^ When adjusting for population differences, we predict that more than 4.5 million open fractures occur per year in India. This figure may be an underestimation, given the high population density in the large urban centers in India. These fractures can involve significant morbidity and are inherently worrisome, as the body's protective skin barrier has been broken and the potential for contamination is high. The correct and timely management of these injuries can benefit our patients and lead to more favorable outcomes.

When deciding on the treatment strategy, the treating surgeon must consider the patient's condition, the mechanism of injury, and the fracture type. Although some of the most impressive injury patterns are from high-energy mechanisms, more commonly, patients present with an open fracture from a simple low-energy mechanism such as a fall. Each fracture could conceivably be treated quite differently, ranging from external fixation and delayed closure or fixation to immediate irrigation, debridement, and primary closure. The status of the soft tissues surrounding the fracture site is of paramount importance in this decision-making process, which usually influences the initial management.

Goals of open fracture management are well known and include the prevention of infection, achievement of bony union, and the restoration of function. Current treatment strategies in the care of open fractures are continuously studied, improved, and adjusted as our literature base expands. Important principles include antibiotic utilization, timing of initial surgical intervention, type of wound closure, antibiotic delivery methods, tetanus coverage, wound irrigation, and adjunctive therapies to assist with fracture union. This review aims to provide current information and references for further reading on these topics and provide a framework for decision-making when presented with an open fracture in the acute setting.

## CLASSIFICATION SYSTEMS

The purpose of any fracture classification system in the clinical setting is to allow communication that infers fracture morphology and treatment parameters. In the setting of open fractures, there are two classification systems that surgeons treating these injuries should be familiar with. They are the Gustilo classification and the Mangled Extremity Severity Scale (MESS).^5^–^8^ The Gustilo classification has been the most widely used system and is generally accepted as the primary classification system for open fractures. This system takes into consideration the energy of the fracture, soft-tissue damage, and the degree of contamination. It has been modified since the original classification to allow a more accurate prognosis for more severe injuries (i.e., Type III injuries).^6^,^9^ There has been some concern in the literature regarding the interobserver reliability of this system.^10^ In this study, the surgeons interpreted color movies of examinations and radiographs of patients and then classified the injuries based on that screening. Overall, they demonstrated 60% agreement. We contend that classification of the injury should be made in the operating room at the conclusion of the initial irrigation and debridements (see Table 1 for details).

**Table 1:** Gustilo open fracture classification system^5^,^6^

Gustilo type	Definition	Example fracture patterns
I	Open fracture, clean wound, wound <1 cm in length	Simple transverse or short oblique fractures
II	Open fracture, wound > 1 cm in length without extensive soft-tissue damage, flaps, avulsions	Simple transverse or short oblique fractures
III	Open fracture with extensive soft-tissue laceration, damage, or loss or an open segmental fracture. This type also inculdes open fractures caused by farm injuries, fractures requiring vascular repair, or fractures that have been open for 8 h prior to treatment	High energy fracture pattern with significant involvement of surrounding tissues
IIIA	Type III fracture with adequate periosteal coverage of the fracture bone despite the extensive soft-tissue laceration or damage	Gunshot injuries or segmental fractures
IIIB	Type III fracture with extensive soft-tissue loss and periosteal stripping and bone damage. Usually associated with massive contamination. Will often need further soft-tissue coverage procedure (i.e. free or ratational flap)	Above patterns but usually very contaminated
IIIC	Type III fracture associated with an arterial injury requiring repair, irrespective of degree of soft-tissue injury.	Above patterns but with vascular injury needing repair

The second classification system, the MESS, was originally designed as an objective tool to assist the surgeon in decision-making for amputation *vs* limb salvage in complex lower extremity trauma.^8^ This rating scale takes into consideration the skeletal and soft tissue damage, limb ischemia time, presence of shock, and the patient's age. Multiple studies have examined the efficacy of the MESS both retrospectively and prospectively and have found it to correlate well with the treatment of major limb trauma. It has been suggested that a score of greater than or equal to 7 is predictive of amputation with nearly 100% accuracy [Table 2].^8^,^11^–^19^

**Table 2:** The mangled extremity severity scale^8^

Component	Point
Skeletal and soft-tissue injury	
Low energy (stab; simple fracture civilian gunshot wound)	1
Medium energy (open or multiple fractures, dislocation)	2
High energy (close-range shotgun or military gunshot wound, crush injury)	3
Very high energy (same as above plus gross contamination, soft tissue avulsion)	4
Limb ischemia (score is doubled for ischemia >6 h)	
Pulse reduced or absent but perfusion normal	1
Pulselessness; paresthesias, diminished capillary refill	2
Cool, paralyzed, insensate, numb	3
Shock	
Systolic blood pressure always >90 mm Hg	0
Hypotensive transiently	1
Persistent hypotension	2
Age (years)	
<30	0
30–50	1
>50	2

More recent information has emerged in the management of severe lower extremity trauma and the effectiveness of the MESS and other lower extremity trauma scoring systems, including the Limb Salvage Index and the Predictive Salvage Index. In one of the largest multicenter, prospective outcome studies pertaining to lower extremity trauma, the Lower Extremity Assessment Project (LEAP), found in a study of more than 500 patients with lower extremity trauma that injury factors with the highest significance in the decision for limb salvage were not solely the measures listed in the above scoring systems. The LEAP group found that the most significant factors were tibial fracture pattern, presence of an open foot fracture, bone loss, muscle injury, vein injury, arterial injury, and the absence of plantar sensation. Severe muscle injury had the highest impact on the surgeon with loss of plantar sensation being second.^20^ More recent follow-up data have challenged the importance of the insensate foot in the decision for amputation.^21^ Interestingly, this study found more than one-half of the patients presenting with an insensate foot treated with reconstruction ultimately regained sensation in two years.

The decision for amputation or limb salvage should be made with careful consideration of multiple factors to include not only the MESS parameters and those from the LEAP study, but also the emotional impact, social impact, and psychological recovery necessary for physical recovery.^20^,^22^ The assistance of the patient and their family is also encouraged. We ultimately advise initial surgical exploration prior to decision-making in the severely injured extremity.^17^

## CONTAMINATION OF OPEN FRACTURE AND USE OF ANTIBIOTICS

All open fractures are by definition contaminated and must be treated as such. The treatment methods may differ depending on the type of fracture. Infection risks also differ by fracture type and have been reported to be ranging from 0 to 2% for Type I fractures, 2 to 10% for Type II fractures, and 10 to 50% for Type III fractures.^5^,^9^,^23^ More recent studies have shown that the rates of clinical infection increased to 1.4% (7/497) for Type I fractures, 3.6% (25/695) for Type II fractures, and to 22.7% (45/198) of Type III fractures.^24^ These data are similar to a more recent study on the treatment of open tibia fractures.^25^

Antibiotic treatment with open fracture management should be automatic with early administration being paramount [Table 3], ideally within 3 h of injury. The risk of infection has been shown to decrease six-fold with this practice.^24^,^26^ With the propensity for gram-positive infections with Type I and II fractures, a first-generation cephalosporin is generally recommended. Some authors have advocated adding gram-negative coverage as well.^24^,^25^,^27^ Type III fractures often have contamination from gram-negative organisms, and in the case of soil-contaminated wounds (i.e., farm injuries), additional coverage should be added for anaerobic bacteria. Typically, this would include penicillin for the risk of a Clostridial infection. In the treatment of open fractures in the hospital setting, the surgeon must also be concerned for nosocomial infections, namely by *Staphylococcus aureus* and aerobic gram-negative bacilli such as *Pseudomonas*.^25^ Specific antibiotic coverage for these organisms may be indicated. The duration of antibiotic therapy in the treatment of open fractures has been suggested to be between 1 and 3 days without any solid agreement on a firm end point.^25^,^26^,^28^–^30^ We typically maintain antibiotic coverage until the wound is closed. Our recommended treatment regimen is detailed in Table 3.

**Table 3:** Recommendations for antibiotic therapy in open fracture management (all medicine to be given intravenously)

Fracture type	Clinical infection rates %^24^	Antibiotic choice	Antibiotic duration
I	1.4	Cefazolin*	Every 8 h for three doses
II	3.6	Pipercacillin/tazobactam† OR Cefazolin and tobramycin	Continue for 24 h after wound closure
IIIA	22.7	Pipercacillin/tazobactam OR Cefazolin AND tobramycin‡ plus penicillin for anaerobic bacteria if needed	Three days
IIIB	10-50	Pipercacillin/tazobactam OR Cefazolin AND tobramycin plus penicillin§ for anaerobic bacteria if needed	Continue for three days after wound closure
IIIC	10-50	Pipercacillin/tazobactam OR Cefazolin AND tobramycin plus penicillin for anaerobic bacteria if needed	Continue for three days after wound closure

* 1-2 g intravenously (IV) every 8 h

† 3.375 g IV every 6 h

‡ 5.1 mg/kg IV every 24 h (recommend pharmacy to assist with monitoring levels)

§ 2–4 million units IV every 4 h

**Figure 1:**
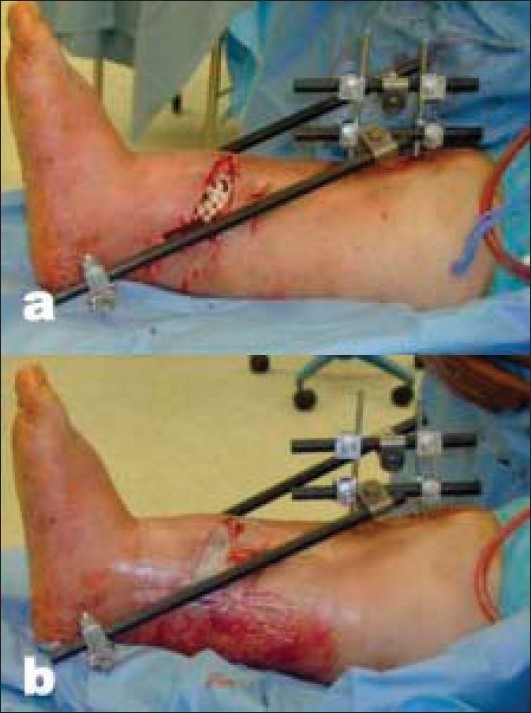
(a) Clinical photograph of a open fracture leg shows antibiotic bead pouch before occlusive dressing application. (b) Antibiotic bead pouch with occlusive dressing applied

Local antibiotic delivery must be considered when extensive contamination is present. This is commonly done with an “antibiotic bead-pouch” construct formed with antibiotic powder and polymethylmethacrylate (PMMA) cement. These constructs are available commercially or can also be easily made in the operating room with readily available equipment. A recommended technique we follow includes forming beads over 24-gauge wire with 3.6 g of tobramycin mixed with 40 g of PMMA cement.^31^ The beads are counted and then placed into the wound and covered with an impermeable dressing (i.e., Ioban, 3M, Minneapolis, MN). This simple technique when used in conjunction with systemic antibiotics has been shown to decrease infection rates from 12 to 3.7% in severe open fractures [Figure 1].^32^ At our institution, this bead-pouch technique is occasionally used after preliminary debridement when surgical plans dictate a return to the operating room within 48 h for further debridement.

Wound contamination with dirt, saliva, or feces; puncture wounds, including unsterile injections; missile injuries; burns; frostbite; avulsions; and crush injuries must raise concerns for *Clostridium tetani*, the anaerobic gram-positive bacterial species responsible for tetanus.^33^ Prophylaxis and treatment for tetanus should be considered for every patient with an open fracture. In the United States, the Centers for Disease Control and Prevention recommend tetanus immunization via tetanus toxoids at 2, 4, and 6 months, 12–18 months, 5 years, 11–12 years, and then at 10-year intervals for maintenance immunization. Any patient presenting with an open fracture who has not completed the tetanus toxoid immunization or has not had their booster in the last 5 years should be given a tetanus toxoid booster. If the wound is prone to contamination with *Clostridium tetani*, the tetanus toxoid should be combined with 250–500 IU of human tetanus immune globulin (HTIG). Furthermore, if more than 10 years has elapsed since the last tetanus booster or the patient's immune system is compromised, both the tetanus toxoid and HTIG should be given.^33^ The HTIG will offer most patients 3 weeks of protection [Table 4].

**Table 4:** *Clostridium tetani* prophylaxis recommendations^33^

Tetanus immunization status	Recommended dosing
Tetanus booster within last 5 years necessary	No further treatment
More than 5 years since booster or has not completed immunization series	Tetanus toxoid (if wound tetanus prone, give HTIG)
More than 10 years since booster or immune system compromised	Tetanus toxoid and HTIG

HTIG: Human tetanus immune globulin.

## INITIAL SURGICAL DEBRIDEMENT

The timing of initial surgical intervention has wide variance within the literature. Historically, the 6-hour rule has been employed as the time limit within which an open fracture should be taken to the operating room for initial debridement.^5^ Many factors influence this parameter including the operating room availability, surgeon availability, and the patient's physiologic status.^34^ Challenges can arise when striving to adhere to this time limit including operating under conditions that are less than ideal (i.e., nonorthopedic surgical teams, poor implant availability, surgeon and personnel fatigue, etc.). This unfortunately can result in adverse events with patient outcomes. The optimal environment for surgical care of the orthopedic trauma patient involves surgical teams that are well-rested and experienced with the procedures being performed. Strict adherence to the emergent 6-h rule does not seem to be justified based on empiric evidence available in the literature.^34^–^44^

Open fractures should be taken to the operating room in an urgent manner using appropriate surgical judgment. There are certain scenarios when more emergent debridements may be needed. These may include Type III injuries with vascular injury and/or gross fecal or soil contamination. If surgery for an open fracture is to be delayed, temporizing treatment should include sterile and antiseptic coverage (i.e., with Technicare soap solution or iodine-derivative) and provisional splinting with attention paid to basic length, rotation, and alignment. A preliminary fracture reduction may need to be performed in the emergency room. Once the wound is dressed and splinted, the covering should not be lifted until the patient is delivered to the operating room as this practice can increase the infection rate by a factor of 3–4.^45^–^46^ Ideally, a digital photograph can be taken at the initial evaluation and used for further communication between providers. We find this to be especially helpful in academic trauma centers where residents and fellows initially evaluate the patients and communicate findings to the attending surgeon.

## IRRIGATION AND DEBRIDEMENT PRINCIPLES

Perhaps the most important aspect in the treatment of open fractures is the initial surgical intervention with irrigation and meticulous debridement of the injury zone. In fact, we believe that the surgeon should spend as much time for planning and performing the debridement as for the fixation of the fracture. This initial debridement should include a sequential evaluation of skin, fat, fascia, muscle, and bone [Table 5]. The propensity to excise as little possible should be avoided in open fracture management given the relatively high contamination rate of these injuries, especially in Type III injuries.^6^ Our approach with open fracture management is to remove any obvious devitalized tissue (including bone) at the initial debridement. If a second debridement is warranted, some questionable muscle may be left until the next scheduled debridement. Ideally, coverage of the open fracture should take place after one to two formal debridements.^23^ One of the most important assessments in the debridement process is vascularity to the affected tissues. This applies not only to excision of devascularized tissues but also to the extension of the open fracture wound through uninterrupted skin. Knowledge of angiosomes and attention to their patterns can help with avoiding wound-healing complications. Incisions are optimally placed between angiosomes to prevent devascularizing portions of the wound.^47^–^49^

**Table 5:** Debridement principles in open fracture management

Tissue	Principles
Skin	Excise all devitalized skin and resect edges until dermal bleeding is encountered. Extend the open wound to evaluate underlying structures. Longitudinal incisions are best.
Subcutaneous tissue and fat	Excise all devitalized tissue. Affected subcutaneous fat and tissue should be freely excised. These tissues have a sparse blood supply and on subsequent debridements, further devitalization may become apparent.
Fascia	Excise all devitalized tissue. As with subcutaneous fat, contaminated fascia should be freely excised. It is vital to recall that compartment syndromes can still occur in the face of open fractures and complete compartment releases should be undertaken if compartment syndrome is suspected.
Muscle	Excise all devitalized tissue. Muscle provides an excellent environment for bacteria to flourish. Thus, extensive debridement of contaminated and devascularized tissue should be completed. Attention to the classic “C’s” of muscle viability can assist the decision for excision: color, consistency, contractility, and capacity to bleed. Caution should be taken with excision of tendons and ligaments. These should be meticulously cleaned and left for later debridement if they prove to be devitalized.
Bone	Remove all devitalized bone. The ends of the bone should be delivered into the wound and cleaned/debrided. Devitalized fragments of bone should be removed. Large portions of cancellous bone can be cleaned and used as graft material (only if not directly involved in the open fracture environment and not grossly contaminated. Clinical judgment is needed in this case).

Irrigation, along with debridement, is absolutely crucial in the management of open fractures. The removal of contaminating debris and the decrease of potentially infective bacterial loads decrease the chances of acute and chronic infections. Our institution uses a popular protocol that calls for 3 L for a Type I open fracture, 6 L for a Type II open fracture, and 9 L for a Type III open fracture [Table 6].^50^ Surgeons should favor using a low to medium pressure lavage device as higher-pressure devices have been associated with added tissue or bone damage.^51^–^52^ Alternatively, if power irrigation is not available, bulb irrigation may be sufficient. Additives to irrigation have remained a source of controversy. Their use is based mostly on anecdotal reports of beneficial outcomes or avoidance of complications. Recently, a Level I evidence study found that there is no significant difference between antibiotic and liquid castile soap solutions (Triad Medical, Franklin, Wisconsin) in wound infection or bone-healing rates in the management of open fractures. Interestingly, this same study also found a statistically significant link between wound-healing problems and antibiotic (bacitracin) irrigation.^53^ Overall, there is a lack of evidence-based recommendations in the literature to guide surgeons on the appropriate additives for irrigations.

**Table 6:** Irrigation principles in the open fracture management

Gustilo fracture type	Irrigation volume/additives
I	3 L normal saline with liquid castile soap additive only. Alternatively, no additive may be used.
II	6 L normal saline with liquid castile soap additive only.
IIIA-C	9 L normal saline with liquid castile soap additive. Highly contaminated wounds may benefit from antibiotic in the irrigation solution.

## TIMING OF WOUND CLOSURE

Options for wound closure in the treatment of open fractures include primary closure of the skin, split-thickness skin-grafting, and the use of either free or local muscle flaps. The timing of open wound closure has proponents in the immediate, early, and delayed categories. Although these terms are used frequently in the literature, their use has yet to gain universal acceptance.^54^ Traditionally, immediate closure is defined as wound closure at the time of the initial surgical intervention. Early closure is within the 24–72 h window, and delayed or late closure extends beyond 3 days. Historically, surgeons have opted to delay closure because of the perceived risks of clostridial infections and gas gangrene. This concern is certainly present in the grossly contaminated open fracture. Current treatment strategies correctly emphasize the importance of debridement and irrigation, and adhering to these principles has allowed surgeons to consider earlier closure and immediate primary closure in some cases when certain criteria are met. These have been suggested to include debridement performed within 12 h, no excess skin loss primarily or secondarily during debridement, skin approximation possible without tension, no gross soil or other similar contamination, and no vascular insufficiency.^55^ Recent studies have shown that open fractures are often contaminated with nosocomial organisms (i.e., *Pseudomonas*) and that early closure may help prevent these infections.^25^,^56^–^61^ Several studies have examined immediate closure of open tibia fractures and have documented that this practice resulted in decreased infection rates, decreased reoperations, and decreased time to bony union.^37^,^62^–^64^

**Figure 2:**
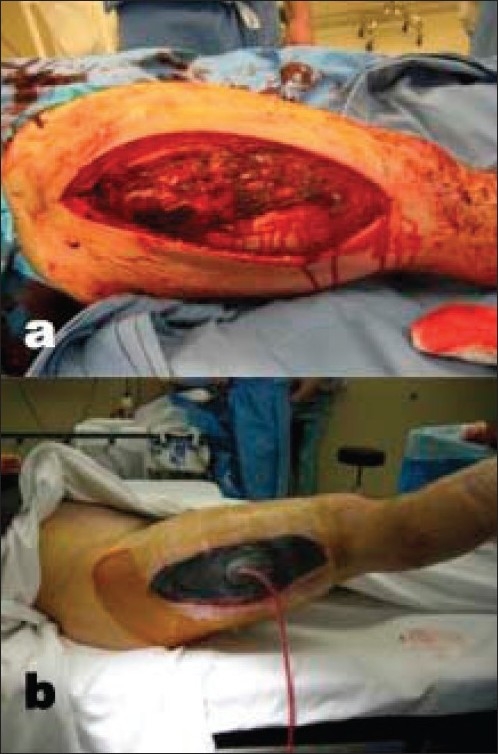
(a) Clinical photograph of thigh shows Open wound prior to wound vacuum dressing. (b) Open wound appearance after wound vacuum dressing

Our recommendation is toward primary closure of Type I, Type II, and a few selected Type IIIA fractures. The most important factors in our decision-making process is the adequacy of the initial debridement and the degree of wound contamination. If there is any doubt regarding the safety of primary closure, we opt to wait until the second surgical debridement and make further treatment decisions at that time. If a primary closure is conducted and there is questionable tissue viability noted postoperatively, we have a very low threshold for reopening the wound 48–72 h after initial closure. If possible, we aim to have coverage completed within 72 h preferably with primary closure. Particular attention must be paid to tension across the wound closure site. Tension may interfere with wound healing by decreasing the vascularity across the incision. Close relationships with plastic and tissue reconstructive teams can facilitate early closure if flap coverage is necessary. A valuable adjunct to wound closure has been the wound vacuum-assisted closure device (VAC; KCI, San Antonia, TX).^65^–^69^ It has been shown that this device aids in wound healing by reducing edema, enhancing granulation tissue formation, and increasing local blood flow [Figure 2].^70^,^71^ We utilize this vacuum-assisted closure concept often when immediate closure is not possible although it is important to realize that this method does not necessarily reduce infection rates or allow a permissible delay in wound closure.^69^,^72^ The choice between the wound vacuum-assisted closure device and the antibiotic bead-pouch depends on the degree of wound contamination and surgeon preference.

## SKELETAL STABILIZATION

Early stabilization of open fractures provides many benefits to the injured patient. It protects the soft tissues around the zone of injury by preventing further damage from mobile fracture fragments. It also restores length, alignment, and rotation—all vital principles of fracture fixation. This restoration of length also helps decrease soft tissue dead spaces and has been shown in studies to decrease the rates of infection in open fractures.^40^,^73^,^74^ Lastly, early fixation allows improved access to soft tissues surrounding the injury and facilitates the patient's early return to normal function.^75^ The surgeon has many choices when deciding on fixation constructs: skeletal traction, external fixation, and intramedullary nails and plates. The choice of fixation involves the bone fractured and the fracture location (intraarticular, metaphyseal, diaphyseal), the extent of the soft-tissue injury and the degree of contamination, and the physiologic status of the patient.^23^ There are also certain situations in which more than one method may be used (i.e., fibula plating of a Pilon fracture in which fibula fixation helps restore length and rotation in conjunction with an external fixator placed across the tibial-talar joint).

Skeletal traction and external fixation are the quickest fixation constructs to employ. The use of skeletal traction should be reserved only for selected open fracture types (i.e., pelvis fractures and very proximal femur fractures) and if used, it should only be for a short selected time. External fixation is a valuable tool in the surgeon's arsenal for acute open fracture management. Indications for external fixation are grossly contaminated open fractures with extensive soft-tissue compromise, the Type IIIA-C injuries, and when immediate fixation is needed for physiologically unstable patients. This later indication involves the damage control concept of orthopedic trauma.^76^–^79^ When not being used for definitive fixation, external fixation is placed as a spanning construct leaving the zone of injury free of pins and easily accessible for imaging studies and future fixation. The surgeon should also be cognizant of future incision placement and avoid placing external fixation pins in these areas.

Plate fixation is generally indicated for open upper extremity fractures and periarticular fractures where reconstruction of the articular surface is paramount. The exception to early periarticular fixation is when a staged protocol is being used for extensive articular and soft-tissue involvement.^80^,^81^ Higher infections rates have been reported with plate fixation of open fractures,^82^,^83^ so diligence is needed when the decision is made to use plates. Current plating technology and less-invasive techniques are lowering these rates and providing patients with good to excellent results.^84^–^88^

Intramedullary nail fixation remains the mainstay of treatment for most open tibial shaft fractures and for selected femoral fractures. A recent study showed that more than 88% of surgeons use an intramedullary nail for open Type I and II tibial shaft fractures. Interestingly, this number decreases to 68% for Type IIIA and to 48% for Type IIIB fractures. The choice in the latter is external fixation.^89^ There has been considerable debate in the literature regarding reamed and nonreamed intramedullary nails with proponents for both methods. In an effort to answer this question, one of the largest studies in orthopedic trauma surgery was recently completed.^90^ The Study to Prospectively evaluate Reamed Intramedullary Nails in Tibial fractures (SPRINT) enrolled more than 1300 patients and randomized them to reamed or nonreamed tibial nails. There were 400 open fractures enrolled in the study, and the major end point was reoperation. They found a 27% risk of revision in open fractures, regardless of the treatment used. Although not statistically significant, a trend was noted toward the need for revision surgery SPRINT (*P* = 0.16) when reamed nails were used in open fractures. It is important to note that study design did not allow any reoperations within 6 months of the index procedure.^90^ We continue to promote this recommendation at our institutions.

Conversion from external fixation to an intramedullary nail has received considerable attention in the literature. Original reporting of this conversion had alarming results with infection and nonunion rates of 44 and 50%, respectively.^91^ Subsequent studies have demonstrated better results.^92^–^95^ Conclusions from these studies seem to indicate that conversion from external fixation to an intramedullary nail is safe given two parameters: conversion in less than 2 weeks and absence of pin site infections. Conversion after pin site infections may require additional time and antibiotic treatment after removing the external fixator and placement of the intramedullary nail.^92^ We use this conversion frequently for complex trauma and Type III open fractures.

## ADJUNCTIVE THERAPIES

An inherent risk in the treatment of open fractures is the occurrence of a nonunion. This is typically defined as a lack of osseous union across three cortices as seen by radiographs 9 months postoperatively. This risk has been well quantified in the literature, especially with regard to open tibia fractures, with rates ranging from 5% for Type I open fractures and 18–38% for Type IIIA-C fractures.^62^,^96^–^100^ This statistic is not surprising in that the open fractures release their valuable fracture hematoma through the fracture site, which drastically reduces the concentration of valuable postinjury healing factors. There has been intense study into adjunctive therapies to assist the surgeon on the management and prevention of nonunions. With open fracture management, adjunctive therapies include prophylactic bone grafting and the application of bone morphogenic proteins (BMPs) at the initial operation.

In the largest study to date using BMPs, the BMP-2 Evaluation in Surgery for Tibia Trauma (BESST) trial, demonstrated that BMP-2 can be used safely in open fractures. In the study of 145 open tibia fractures, there was a 44% reduction in secondary surgeries.^101^ Certainly not all fractures warrant BMP application, and the high costs associated with BMPs play a major role in its utilization. At our institution, the use of BMPs in acute fracture management is limited to selected Gustilo Type III tibia fractures in patients with significant comorbidities that may impede fracture healing (diabetes, tobacco use, etc.).^102^

Prophylactic bone grafting can also be used in the early treatment of open fractures. The literature has several examples of studies pertaining to immediate or early prophylactic bone grafting, and this practice has reported to shorten the time to fracture union and reduce the rate of delayed union by more than 11 weeks.^103^–^106^ The utilization of prophylactic bone grafting is not routine at our hospital, and we do not typically intervene before 6 months postoperatively.

## CONCLUSIONS

The above review provides a framework that the surgeon can reference when treating patients with open fractures. The management of open fractures involves the adherence to principles discussed earlier. Using a principle-based treatment regimen can help improve patient outcomes while avoiding complications and adverse events. Ultimately, this is the surgeon's goal, and patients will benefit from the early return to normal function.

## References

[1] Praemer A, Furner S, Rice DP (1992). Musculoskeletal conditions in the United States.

[2] (2008). Information about Orthopaedic Patients and Conditions - AAOS.

[3] Court-Brown CM, McQueen MM, Quaba AA (1996). Management of open fractures.

[4] Court-Brown CM, Rimmer S, Prakash U, McQueen MM (1998). The epidemiology of open long bone fractures. Injury.

[5] Gustilo RB, Anderson JT (1976). Prevention of infection in the treatment of one thousand and twenty-five open fractures of long bones: Retrospective and prospective analyses. J Bone Joint Surg Am.

[6] Gustilo RB, Mendoza RM, Williams DN (1984). Problems in the management of type III (severe) open fractures: A new classification of type III open fractures. J Trauma.

[7] Tscherne H, Oestern HJ (1982). A new classification of soft-tissue damage in open and closed fractures. Unfallheilkunde.

[8] Johansen K, Daines M, Howey T, Helfet D, Hansen ST (1990). Objective criteria accurately predict amputation following lower extremity trauma. J Trauma.

[9] Gustilo RB, Gruninger RP, Davis T (1987). Classification of type III (severe) open fractures relative to treatment and results. Orthopedics.

[10] Brumback RJ, Jones AL (1994). Interobserver agreement in the classification of open fractures of the tibia: The results of a survey of two hundred and forty-five orthopaedic surgeons. J Bone Joint Surg Am.

[11] Helfet DL, Howey T, Sanders R, Johansen K (1990). Limb salvage versus amputation: Preliminary results of the Mangled Extremity Severity Score. Clin Orthop Relat Res.

[12] Slauterbeck JR, Britton C, Moneim MS, Clevenger FW (1994). Mangled extremity severity score: An accurate guide to treatment of the severely injured upper extremity. J Orthop Trauma.

[13] Durham RM, Mistry BM, Mazuski JE, Shapiro M, Jacobs D (1996). Outcome and utility of scoring systems in the management of the mangled extremity. Am J Surg.

[14] O'Sullivan ST, O'Sullivan M, Pasha N, O'Shaughnessy M, O'Connor TP (1997). Is it possible to predict limb viability in complex Gustilo IIIB and IIIC tibial fractures? A comparison of two predictive indices. Injury.

[15] Fagelman MF, Epps HR, Rang M (2002). Mangled extremity severity score in children. J Pediatr Orthop.

[16] Sharma S, Devgan A, Marya KM, Rathee N (2003). Critical evaluation of mangled extremity severity scoring system in Indian patients. Injury.

[17] Elsharawy MA (2005). Arterial reconstruction after mangled extremity: Injury severity scoring systems are not predictive of limb salvage. Vascular.

[18] Togawa S, Yamami N, Nakayama H, Mano Y, Ikegami K, Ozeki S (2005). The validity of the mangled extremity severity score in the assessment of upper limb injuries. J Bone Joint Surg Br.

[19] Rush RM, Kjorstad R, Starnes BW, Arrington E, Devine JD, Andersen CA (2007). Application of the Mangled Extremity Severity Score in a combat setting. Mil Med.

[20] MacKenzie EJ, Bosse MJ, Kellam JF, Burgess AR, Webb LX, Swiontkowski MF (2002). Factors influencing the decision to amputate or reconstruct after high-energy lower extremity trauma. J Trauma.

[21] Bosse MJ, McCarthy ML, Jones AL, Webb LX, Sims SH, Sanders RW (2005). The insensate foot following severe lower extremity trauma: An indication for amputation?. J Bone Joint Surg Am.

[22] Dougherty PJ (2007). Open tibia fracture: Amputation versus limb salvage, Opinion: below-the-knee amputation. J Orthop Trauma.

[23] Zalavras CG, Marcus RE, Levin LS, Patzakis MJ (2007). Management of open fractures and subsequent complications. J Bone Joint Surg Am.

[24] Patzakis MJ, Wilkins J (1989). Factors influencing infection rate in open fracture wounds. Clin Orthop Relat Res.

[25] Templeman DC, Gulli B, Tsukayama DT, Gustilo RB (1998). Update on the management of open fractures of the tibial shaft. Clin Orthop Relat Res.

[26] Patzakis MJ, Wilkins J, Moore TM (1983). Considerations in reducing the infection rate in open tibial fractures. Clin Orthop Relat Res.

[27] Patzakis MJ, Wilkins J, Moore TM (1983). Use of antibiotics in open tibial fractures. Clin Orthop Relat Res.

[28] Dellinger EP, Caplan ES, Weaver LD, Wertz MJ, Droppert BM, Hoyt N (1988). Duration of preventive antibiotic administration for open extremity fractures. Arch Surg.

[29] Dellinger EP, Miller SD, Wertz MJ, Grypma M, Droppert B, Anderson PA (1988). Risk of infection after open fracture of the arm or leg. Arch Surg.

[30] Zalavras CG, Patzakis MJ (2003). Open fractures: Evaluation and management. J Am Acad Orthop Surg.

[31] Zalavras CG, Patzakis MJ, Holtom P (2004). Local antibiotic therapy in the treatment of open fractures and osteomyelitis. Clin Orthop Relat Res.

[32] Ostermann PA, Seligson D, Henry SL (1995). Local antibiotic therapy for severe open fractures. A review of 1085 consecutive cases. J Bone Joint Surg Br.

[33] Bleck TP, Mandell G, Douglas R, Bennett J (2005). Clostridum tetani (Tetanus). Principles and Practice of Infectious Diseases.

[34] Pollak AN (2006). Timing of debridement of open fractures. J Am Acad Orthop Surg.

[35] Ashford RU, Mehta JA, Cripps R (2004). Delayed presentation is no barrier to satisfactory outcome in the management of open tibial fractures. Injury.

[36] Bednar DA, Parikh J (1993). Effect of time delay from injury to primary management on the incidence of deep infection after open fractures of the lower extremities caused by blunt trauma in adults. J Orthop Trauma.

[37] Gopal S, Majumder S, Batchelor AG, Knight SL, De Boer P, Smith RM (2000). Fix and flap: The radical orthopaedic and plastic treatment of severe open fractures of the tibia. J Bone Joint Surg Br.

[38] Harley BJ, Beaupre LA, Jones CA, Dulai SK, Weber DW (2002). The effect of time to definitive treatment on the rate of nonunion and infection in open fractures. J Orthop Trauma.

[39] Khatod M, Botte MJ, Hoyt DB, Meyer RS, Smith JM, Akeson WH (2003). Outcomes in open tibia fractures: Relationship between delay in treatment and infection. J Trauma.

[40] Merritt K (1988). Factors increasing the risk of infection in patients with open fractures. J Trauma.

[41] Sungaran J, Harris I, Mourad M (2007). The effect of time to theatre on infection rate for open tibia fractures. ANZ J Surg.

[42] Reuss BL, Cole JD (2007). Effect of delayed treatment on open tibial shaft fractures. Am J Orthop.

[43] Crowley DJ, Kanakaris NK, Giannoudis PV (2007). Debridement and wound closure of open fractures: The impact of the time factor on infection rates. Injury.

[44] Webb LX, Bosse MJ, Castillo RC, MacKenzie EJ (2007). LEAP Study Group; Analysis of surgeon-controlled variables in the treatment of limb-threatening type-III open tibial diaphyseal fractures. J Bone Joint Surg Am.

[45] Browner BD (2003). Skeletal trauma: Basic science, management, and reconstruction.

[46] Tscherne H, Gotzen L (1984). Fractures with soft tissue injuries.

[47] Taylor GI, Palmer JH (1987). The vascular territories (angiosomes) of the body: Experimental study and clinical applications. Br J Plast Surg.

[48] Attinger CE, Evans KK, Bulan E, Blume P, Cooper P (2006). Angiosomes of the foot and ankle and clinical implications for limb salvage: Reconstruction, incisions, and revascularization. Plast Reconstr Surg.

[49] Taylor GI (2003). The angiosomes of the body and their supply to perforator flaps. Clin Plast Surg.

[50] Anglen JO (2001). Wound irrigation in musculoskeletal injury. J Am Acad Orthop Surg.

[51] Bhandari M, Schemitsch EH, Adili A, Lachowski RJ, Shaughnessy SG (1999). High and low pressure pulsatile lavage of contaminated tibial fractures: An in vitro study of bacterial adherence and bone damage. J Orthop Trauma.

[52] Bhandari M, Thompson K, Adili A, Shaughnessy SG (2000). High and low pressure irrigation in contaminated wounds with exposed bone. Int J Surg Investig.

[53] Anglen JO (2005). Comparison of soap and antibiotic solutions for irrigation of lower-limb open fracture wounds: A prospective, randomized study. J Bone Joint Surg Am.

[54] Levin LS (2007). Early versus delayed closure of open fractures. Injury.

[55] Rajasekaran S (2007). Early versus delayed closure of open fractures. Injury.

[56] Carsenti-Etesse H, Doyon F, Desplaces N, Gagey O, Tancrede C, Pradier C (1999). Epidemiology of bacterial infection during management of open leg fractures. Eur J Clin Microbiol Infect Dis.

[57] Byrd HS, Spicer TE, Cierney G (1985). Management of open tibial fractures. Plast Reconstr Surg.

[58] Cierny G, Byrd HS, Jones RE (1983). Primary versus delayed soft tissue coverage for severe open tibial fractures: A comparison of results. Clin Orthop Relat Res.

[59] Caudle RJ, Stern PJ (1987). Severe open fractures of the tibia. J Bone Joint Surg Am.

[60] Fischer MD, Gustilo RB, Varecka TF (1991). The timing of flap coverage, bone-grafting, and intramedullary nailing in patients who have a fracture of the tibial shaft with extensive soft-tissue injury. J Bone Joint Surg Am.

[61] Sinclair JS, McNally MA, Small JO, Yeates HA (1997). Primary free-flap cover of open tibial fractures. Injury.

[62] DeLong WG, Born CT, Wei SY, Petrik ME, Ponzio R, Schwab CW (1999). Aggressive treatment of 119 open fracture wounds. J Trauma.

[63] Hertel R, Lambert SM, Muller S, Ballmer FT, Ganz R (1999). On the timing of soft-tissue reconstruction for open fractures of the lower leg. Arch Orthop Trauma Surg.

[64] Godina M (1986). Early microsurgical reconstruction of complex trauma of the extremities. Plast Reconstr Surg.

[65] Herscovici D, Sanders RW, Scaduto JM, Infante A, Di Pasquale T (2003). Vacuum-assisted wound closure (VAC therapy) for the management of patients with high-energy soft tissue injuries. J Orthop Trauma.

[66] Tarkin IS, Clare MP, Marcantonio A, Pape HC (2008). An update on the management of high-energy pilon fractures. Injury.

[67] Hardwicke J, Paterson P (2006). A role for vacuum-assisted closure in lower limb trauma: A proposed algorithm. Int J Low Extrem Wounds.

[68] Parrett BM, Matros E, Pribaz JJ, Orgill DP (2006). Lower extremity trauma: Trends in the management of soft-tissue reconstruction of open tibia-fibula fractures. Plast Reconstr Surg.

[69] Dedmond BT, Kortesis B, Punger K, Simpson J, Argenta J, Kulp B (2007). The use of negative-pressure wound therapy (NPWT) in the temporary treatment of soft-tissue injuries associated with high-energy open tibial shaft fractures. J Orthop Trauma.

[70] Argenta LC, Morykwas MJ (1997). Vacuum-assisted closure: a new method for wound control and treatment: Clinical experience. Ann Plast Surg.

[71] Morykwas MJ, Argenta LC, Shelton-Brown EI, McGuirt W (1997). Vacuum-assisted closure: A new method for wound control and treatment: animal studies and basic foundation. Ann Plast Surg.

[72] Bhattacharyya T, Mehta P, Smith M, Pomahac B (2008). Routine use of wound vacuum-assisted closure does not allow coverage delay for open tibia fractures. Plast Reconstr Surg.

[73] Worlock P, Slack R, Harvey L, Mawhinney R (1994). The prevention of infection in open fractures: An experimental study of the effect of fracture stability. Injury.

[74] Merritt K, Dowd JD (1987). Role of internal fixation in infection of open fractures: Studies with Staphylococcus aureus and Proteus mirabilis. J Orthop Res.

[75] Gustilo RB, Merkow RL, Templeman D (1990). The management of open fractures. J Bone Joint Surg Am.

[76] Hildebrand F, Giannoudis P, Kretteck C, Pape HC (2004). Damage control: Extremities. Injury.

[77] Pape HC, Hildebrand F, Pertschy S, Zelle B, Garapati R, Grimme K (2002). Changes in the management of femoral shaft fractures in polytrauma patients: From early total care to damage control orthopedic surgery. J Trauma.

[78] Pape HC, Krettek C (2003). Damage control orthopaedic surgery. Unfallchirurg.

[79] Roberts CS, Pape HC, Jones AL, Malkani AL, Rodriguez JL, Giannoudis PV (2005). Damage control orthopaedics: Evolving concepts in the treatment of patients who have sustained orthopaedic trauma. Instr Course Lect.

[80] Sirkin M, Sanders R, DiPasquale T, Herscovici D (2004). A staged protocol for soft tissue management in the treatment of complex pilon fractures. J Orthop Trauma.

[81] Watson JT, Moed BR, Karges DE, Cramer KE (2000). Pilon fractures: Treatment protocol based on severity of soft tissue injury. Clin Orthop Relat Res.

[82] Bach AW, Hansen ST (1989). Plates versus external fixation in severe open tibial shaft fractures: A randomized trial. Clin Orthop Relat Res.

[83] Clifford RP, Beauchamp CG, Kellam JF, Webb JK, Tile M (1988). Plate fixation of open fractures of the tibia. J Bone Joint Surg Br.

[84] Cole PA, Zlowodzki M, Kregor PJ (2004). Treatment of proximal tibia fractures using the less invasive stabilization system: Surgical experience and early clinical results in 77 fractures. J Orthop Trauma.

[85] Fankhauser F, Gruber G, Schippinger G, Boldin C, Hofer HP, Grechenig W (2004). Minimal-invasive treatment of distal femoral fractures with the LISS (Less Invasive Stabilization System): A prospective study of 30 fractures with a follow up of 20 months. Acta Orthop Scand.

[86] Kregor PJ, Stannard JA, Zlowodzki M, Cole PA (2004). Treatment of distal femur fractures using the less invasive stabilization system: Surgical experience and early clinical results in 103 fractures. J Orthop Trauma.

[87] Stannard JP, Wilson TC, Volgas DA, Alonso JE (2003). Fracture stabilization of proximal tibial fractures with the proximal tibial LISS: Early experience in Birmingham, Alabama (USA). Injury.

[88] Syed AA, Agarwal M, Giannoudis PV, Matthews SJ, Smith RM (2004). Distal femoral fractures: Long-term outcome following stabilisation with the LISS. Injury.

[89] Bhandari M, Guyatt GH, Tornetta P, Swiontkowski MF, Hanson B, Sprague S (2002). Current practice in the intramedullary nailing of tibial shaft fractures: An international survey. J Trauma.

[90] Bhandari M (2008). Reamed Versus Non-Reamed Tibial Intramedullary Nail Insertion on Re-operation Rates. 75th Annual Meeting of the American Academy of Orthopaedic Surgeons; 03/2008.

[91] McGraw JM, Lim EV (1988). Treatment of open tibial-shaft fractures: External fixation and secondary intramedullary nailing. J Bone Joint Surg Am.

[92] Nowotarski PJ, Turen CH, Brumback RJ, Scarboro JM (2000). Conversion of external fixation to intramedullary nailing for fractures of the shaft of the femur in multiply injured patients. J Bone Joint Surg Am.

[93] Blachut PA, Meek RN, O'Brien PJ (1990). External fixation and delayed intramedullary nailing of open fractures of the tibial shaft: A sequential protocol. J Bone Joint Surg Am.

[94] Dougherty PJ, Silverton C, Yeni Y, Tashman S, Weir R (2006). Conversion from temporary external fixation to definitive fixation: Shaft fractures. J Am Acad Orthop Surg.

[95] Maurer DJ, Merkow RL, Gustilo RB (1989). Infection after intramedullary nailing of severe open tibial fractures initially treated with external fixation. J Bone Joint Surg Am.

[96] Hope PG, Cole WG (1992). Open fractures of the tibia in children. J Bone Joint Surg Br.

[97] Whorton AM, Henley MB (1998). The role of fixation of the fibula in open fractures of the tibial shaft with fractures of the ipsilateral fibula: Indications and outcomes. Orthopedics.

[98] Stegemann P, Lorio M, Soriano R, Bone L (1995). Management protocol for unreamed interlocking tibial nails for open tibial fractures. J Orthop Trauma.

[99] Bhandari M, Guyatt GH, Swiontkowski MF, Schemitsch EH (2001). Treatment of open fractures of the shaft of the tibia. J Bone Joint Surg Br.

[100] Court-Brown CM, Keating JF, Christie J, McQueen MM (1995). Exchange intramedullary nailing: Its use in aseptic tibial nonunion. J Bone Joint Surg Br.

[101] Govender S, Csimma C, Genant HK, Valentin-Opran A, Amit Y, Arbel R (2002). Recombinant human bone morphogenetic protein-2 for treatment of open tibial fractures: A prospective, controlled, randomized study of four hundred and fifty patients. J Bone Joint Surg Am.

[102] Swiontkowski MF, Aro HT, Donell S, Esterhai JL, Goulet J, Jones A (2006). Recombinant human bone morphogenetic protein-2 in open tibial fractures: A subgroup analysis of data combined from two prospective randomized studies. J Bone Joint Surg Am.

[103] Blick SS, Brumback RJ, Lakatos R, Poka A, Burgess AR (1989). Early prophylactic bone grafting of high-energy tibial fractures. Clin Orthop Relat Res.

[104] Kesemenli CC, Kapukaya A, Subasi M, Arslan H, Necmioglu S, Kayikci C (2004). Early prophylactic autogenous bone grafting in type III open tibial fractures. Acta Orthop Belg.

[105] Kobbe P, Frink M, Oberbeck R, Tarkin IS, Tzioupis C, Nast-Kolb D (2008). Treatment strategies for gunshot wounds of the extremities. Unfallchirurg.

[106] Trabulsy PP, Kerley SM, Hoffman WY (1994). A prospective study of early soft tissue coverage of grade IIIB tibial fractures. J Trauma.

